# Compression and suppression as instances of a similar mechanism affecting tactile perception during movement

**DOI:** 10.3389/fnhum.2015.00217

**Published:** 2015-04-22

**Authors:** Georgiana Juravle

**Affiliations:** Department of Systems Neuroscience, University Medical Center Hamburg-EppendorfHamburg, Germany

**Keywords:** tactile, movement, sensory suppression, time compression, behavior

As we look at the world around us, we make numerous eye-movements, or saccades, toward objects of interest. These eye-movements are rapid/ballistic and most of the time we do not even notice them, not to mention that we are very rarely concerned with their having taken place. Interestingly, convincing psychophysical demonstrations have shown that reliable and intriguing visual phenomena take place around the time of the saccade: For instance, visual stimuli are *suppressed*—That is, we fail to notice them, they are likely to be mislocalized in space along the axis of the saccade, as well as, importantly, they seem to be *compressed* in both space and time (see Burr and Morrone, 2011, for a review). Although all of these less than veridical effects of one's eye-movements might appear worrisome, it is generally accepted that they help the observer by providing a stable world when we move our eyes.

Intriguingly, a similar perceptual distortion has been found to affect tactile perception at the time of movement. For example, we might fail to notice a short tactile tap on our hand when we are reaching for the coffee cup located on the table in front of us. Or when we move, most of the time we might just not feel a tactile tap like a buzzing cellphone as intensely as if the same stimulus was delivered at rest. This phenomenon of tactile suppression, gating, or attenuation has been described for both active and passive movements of the fingers of the hand (e.g., Williams and Chapman, 2002), as well as for other goal-directed arm movements (Buckingham et al., 2010; Juravle et al., 2010; Colino et al., 2014). Similar to the phenomenon of visual saccadic suppression, tactile suppression occurs around the time a movement is initiated (Bays et al., 2005), is affected by the speed of movement (Cybulska-Klosowicz et al., 2011), and by response bias (Juravle and Spence, 2011, 2012; Colino et al., 2014). The tactile suppression occurring before movement onset has been attributed to the generation of the motor command (i.e., efference copy), whereas the attenuation occurring over the execution period of a movement has been primarily explained by a combination of motor command generation and sensory signals resulting from the movement (i.e., sensory re-afference, Chapman and Beauchamp, 2006; Juravle and Spence, 2011); see Figure 1A for a depiction of a typical trial timeline in a tactile suppression experiment.

**Figure 1:**
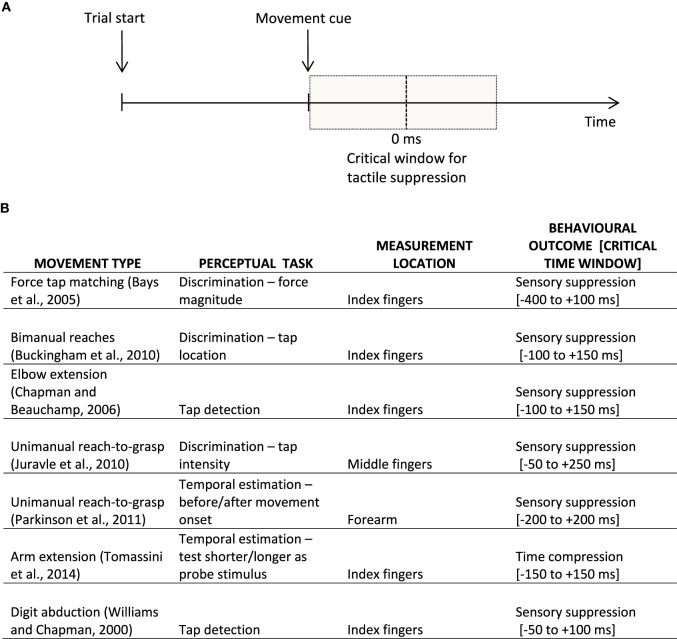
**(A)** Example of a typical trial timeline in a sensorimotor experiment. Participants prepare an instructed movement, which they execute at the appearance of a learned cue. The vertical dotted line signifies movement onset (i.e., participant's reaction time); the shaded gray represents the critical window for sensory suppression. **(B)** Examples of behavioral findings in the movement-related tactile perception literature, split according to the movement type, perceptual task, as well as the location on the body where tactile perception was assessed, together with the behavioral outcome within a rough critical temporal window of appearance; 0 ms is taken to represent the onset of the movement.

Importantly, tactile suppression particularly affects stimulus attributes such as intensity or force (Shergill et al., 2003). However, in addition to these attributes, similar detrimental effects on tactile perception around the time an organism initiates a movement have also been reported in the time domain. For example, the temporal mislocalization of tactile stimuli has been elegantly studied in a paradigm in which participants had to execute a reaching movement toward a predetermined location (Parkinson et al., 2011). In this experiment, participants received a very short tap to either their moving or resting hand. Importantly, this tap could be delivered in a time window of 150 ms prior to or following the (predicted) onset of the movement, a time period when previous research indicates that sensory suppression is maximal (Bays et al., 2005). Participants reported whether they thought the tap was delivered before or after the movement start. When a tap was delivered to a moving hand, it had to be delivered significantly earlier to be perceived as occurring at the same time as the same stimulus delivered to the resting hand. That is, participants perceived the very short tap to have been delivered to their moving limb *later* in time, or as if it had been *delayed* while they had been performing the movement (Parkinson et al., 2011). The authors explained this effect in terms of tactile suppression.

These findings concerning the existence of tactile suppression in the temporal domain were recently explored further in a similar paradigm (Tomassini et al., 2014). The authors had participants execute a simple lateralized movement of the right hand. Following an auditory go signal, a tactile *test* stimulus composed of two very short taps presented in close temporal succession (150 ms) was delivered to either the moving right hand or to the stationary left hand. Adjusting for each participant's mean reaction time to initiate a movement, this tactile test stimulus was delivered so that it fell within a time window of ±200 ms relative to the (predicted) onset of the movement. Once the movement was terminated, a tactile *probe* stimulus composed of two taps with a variable temporal separation (50–250 ms) was delivered to the same hand where the test stimulus had initially been delivered. The participants' task was to compare the temporal length of the test and probe stimuli by reporting which one they believed to be *longer*. In an additional control condition called isometric contraction, the participants pushed their right hand against a block instead of performing the movement—That is, the motor command was still generated for the trial, but the movement was not executed. In this control condition, tactile stimulation was delivered to the right hand only. As expected, the results found distortions in time estimation: When the test stimulus was delivered to the moving hand in a time window ±50 ms from the onset of the movement, participants reported it to be *shorter*, as compared to rightfully acknowledging its duration when the same stimulus was delivered to the non-moving hand. Such a result is in line with the previous finding of tactile suppression in the temporal domain (Parkinson et al., 2011). Moreover, the authors found no difference in temporal estimation between the movement and the isometric conditions, a replication of previous findings on tactile suppression (Post et al., 1994); see Figure 1B for examples of behavioral findings in the movement-related tactile perception literature. When looking at the temporal estimation performance for the moving as opposed to the static hand as a function of the movement onset latency, Tomassini et al. (2014) found that the closer in time to movement onset, the *shorter* the participants' estimations of the time separating the two taps. Furthermore, their results also indicate that the time interval separating two taps delivered to a moving hand is perceived as being significantly *shorter* when participants execute fast movements, as compared to those trials when their movement is slow, particularly shortly before the onset of the planned movement. Such a result is in line with previous research demonstrating that sensory suppression occurs for speeds faster than those typically used in tactile exploration (Cybulska-Klosowicz et al., 2011).

A crucial question that this study raises is whether time compression reflects the same mechanism as sensory suppression, more specifically, whether it would result in a similar behavioral outcome such as tactile suppression. In an attempt to answer this question, Tomassini et al. (2014) conducted an additional experiment. Here, for each participant, they first found the lowest intensity of a single tactile tap detectable during movement. The authors then devised two control conditions which, importantly, were presented to *only* the *resting* right hand. In the first *baseline* condition, the test and the probe stimuli had the same intensity (2.5 V). This means the same physical parameters of tactile stimulation as in the original movement condition were used, with the only difference being that the stimulation was delivered at rest. This first baseline control condition corresponds to the classical control condition encountered in sensory suppression studies. When comparing its results to the performance in the initial movement condition, the typical sensory suppression effect was found, with participants perceiving a shorter temporal separation between the two taps during movement, as compared to the rest baseline. In the second *matched-baseline* control condition, the test stimulus had the lowest detectable intensity during movement, whereas the probe was set at 2.5 V. Here, the authors had two simultaneous manipulations of sensory stimulation, namely different intensities, as well as different temporal separation for the test and probe tap stimuli. The purpose of this second matched-baseline condition was to mimic the movement-related decrement in sensitivity, in a resting hand. When comparing performance in the matched-baseline to the original movement condition, the results highlighted that participants estimated the temporal separation between the two taps as being *shorter* during movement. Such a result was interpreted by the authors to suggest that time compression for tactile stimulation would be independent from tactile suppression. Moreover, having found no difference in behavioral performance between the two control conditions led the authors to ascertain that “the amount of tactile attenuation during movement … is not sufficient to induce any significant bias in apparent time” (Tomassini et al., 2014, p. 9170).

Note, however, that tactile suppression has been traditionally described as a decrement in sensitivity, oftentimes a result of experimentally manipulating the intensity of a particular tactile stimulus. From this view point, the matched-baseline control condition where the decrement in sensitivity is artificially simulated in the absence of movement might lack critical aspects of genuine suppression, and thus be insufficient to rule out sensory suppression as a contributor to temporal compression. The defining aspect of sensory suppression is movement. Consider that typical control experiments for sensory suppression deliver the same intensity of the stimulation both at rest and during movement, with the resulting difference in sensitivity as a marker of sensory suppression. With this important consideration in mind, the decrement found in temporal estimation for the movement condition when compared to the first baseline control condition in Tomassini et al.' study (2014) is already a strong demonstration of the existence of sensory suppression. Importantly, for such an interpretation a unified view of tactile sensory suppression across all physical domains of stimulation (i.e., temporal estimation included) would be needed. I would thus like to argue that by concentrating on the similar decrement in performance observed at a behavioral level for either time or intensity estimation tasks during movement execution in the tactile domain, researchers could advance closer to finding an answer to the ever-paradoxical question of *why* tactile suppression appears at all.

In close connection to the *why* question, a yet unanswered question is whether time compression and sensory suppression *distinctively* contribute to a similar brain function. Before answering this question it must be made sure that the two phenomena of suppression and compression are not the same. That is, in order to acknowledge that time compression is independent from sensory suppression one needs to compare temporal estimation performance under conditions of movement and rest, while keeping the physical intensity of stimulation *constant*. Such an experimental avenue would be possible if one were to eventually investigate different somatic sensibility modalities known not to be affected by sensory suppression, such as nociception or temperature, with, importantly, the acknowledgement of the different physiological pathways the painful/temperature stimulation takes from the movement effector to the brain (Purves et al., 2004). For instance, we already know that painful (Helmchen et al., 2006), as well as thermal stimulation with an external origin (VanDoorn et al., 2005) is not attenuated during self-executed movement. Demonstrating changes in temporal estimation performance between two painful stimuli for conditions of movement and rest could perhaps be the test to pursue in order to argue that compression and suppression separately contribute to a similar brain function. At a methodological level, such an experimental approach of course needs to control for and dissociate the pain and temperature modalities from the tactile stimulation which, inevitably, could accompany them. Note that partly overlapping, yet differential brain activations have been demonstrated for both tactile and painful stimuli (Coghill et al., 1994; Ploner et al., 2000), as well as tactile and thermal stimulation (Davis et al., 1998; Bornhövd et al., 2002; Büchel et al., 2002).

In summary, the question regarding the origin of temporal and spatial distortions in the tactile domain, but also other physical domain distortions still to be found, remains open. Importantly, when looking for the origin of the compression and suppression phenomena, future research needs to consider the perceptual (tactile) changes at the time of movement with regard to their *functional significance* or *relevance* to the organism (Juravle et al., 2013). In this respect, if one was to demonstrate and acknowledge that both tactile suppression and time compression of tactile stimulation have some similar adaptive function for an organism in motion, then both phenomena could be grouped together as serving the same biological function.

## Conflict of interest statement

The author declares that the research was conducted in the absence of any commercial or financial relationships that could be construed as a potential conflict of interest.
